# How Well the Government of Nepal Is Responding to COVID-19? An Experience From a Resource-Limited Country to Confront Unprecedented Pandemic

**DOI:** 10.3389/fpubh.2021.597808

**Published:** 2021-02-17

**Authors:** Binod Rayamajhee, Anil Pokhrel, Gopiram Syangtan, Saroj Khadka, Bhupendra Lama, Lal Bahadur Rawal, Suresh Mehata, Shyam Kumar Mishra, Roshan Pokhrel, Uday Narayan Yadav

**Affiliations:** ^1^School of Optometry and Vision Science, University of New South Wales, Sydney, NSW, Australia; ^2^Department of Infection and Immunology, Kathmandu Research Institute for Biological Sciences, Kathmandu, Nepal; ^3^Central Department of Microbiology, Tribhuvan University, Kathmandu, Nepal; ^4^Shi-Gan International College of Science and Technology, Tribhuvan University, Kathmandu, Nepal; ^5^School of Health Medical and Social Sciences, Central Queensland University, Sydney, NSW, Australia; ^6^Ministry of Health and Population (MoHP), Government of Nepal (GoN), Kathmandu, Nepal; ^7^Department of Microbiology, Institute of Medicine, Tribhuvan University, Kathmandu, Nepal; ^8^Centre for Primary Health Care and Equity, University of New South Wales, Sydney, NSW, Australia; ^9^Centre for Research, Policy and Implementation, Kathmandu, Nepal; ^10^Torrens University, Sydney, NSW, Australia

**Keywords:** covid-19 pandemic, health care delivery, leadership and management, non-communicable diseases, policies and strategies, quarantine management, vaccination

## Abstract

COVID-19, caused by SARS-CoV-2, was first reported in Wuhan, China and is now a pandemic affecting over 218 countries and territories around the world. Nepal has been severely affected by it, with an increasing number of confirmed cases and casualties in recent days, even after 8 months of the first case detected in China. As of 26 November 2020, there were over 227,600 confirmed cases of COVID in Nepal with 209,435 recovered cases and 1,412 deaths. This study aimed to compile public data available from the Ministry of Health and Population (MoHP), Government of Nepal (GoN) and analyse the data of 104 deceased COVID-19 patients using IBM SPSS (Version 25.0). Additionally, this study also aimed to provide critical insights on response of the GoN to COVID-19 and way forward to confront unprecedented pandemic. Figures and maps were created using the Origin Lab (Version 2018) and QGIS (Version 3.10.8). Most of the reported cases were from Bagmati Province, the location of Nepal's capital city, Kathmandu. Among deceased cases, >69% of the patients were male and patients ≥54 years accounted for 67.9% (*n* = 923). Preliminary findings showed respiratory illness, diabetes, and chronic kidney diseases were the most common comorbid conditions associated with COVID-19 deaths in Nepal. Despite some efforts in the 8 months since the first case was detected, the government's response so far has been insufficient. Since the government eased the lockdown in July 2020, Nepal is facing a flood of COVID-19 cases. If no aggressive actions are taken, the epidemic is likely to result in significant morbidity and mortality in Nepal. The best way to curb the effect of the ongoing pandemic in a resource-limited country like Nepal is to increase testing, tracing, and isolation capacity, and to set up quality quarantine centers throughout the nation. A comprehensive health literacy campaign, quality care of older adults and those with comorbidity will also result in the effective management of the ongoing pandemic.

## Overview of the Global Impact of COVID-19

**Figure 1:**
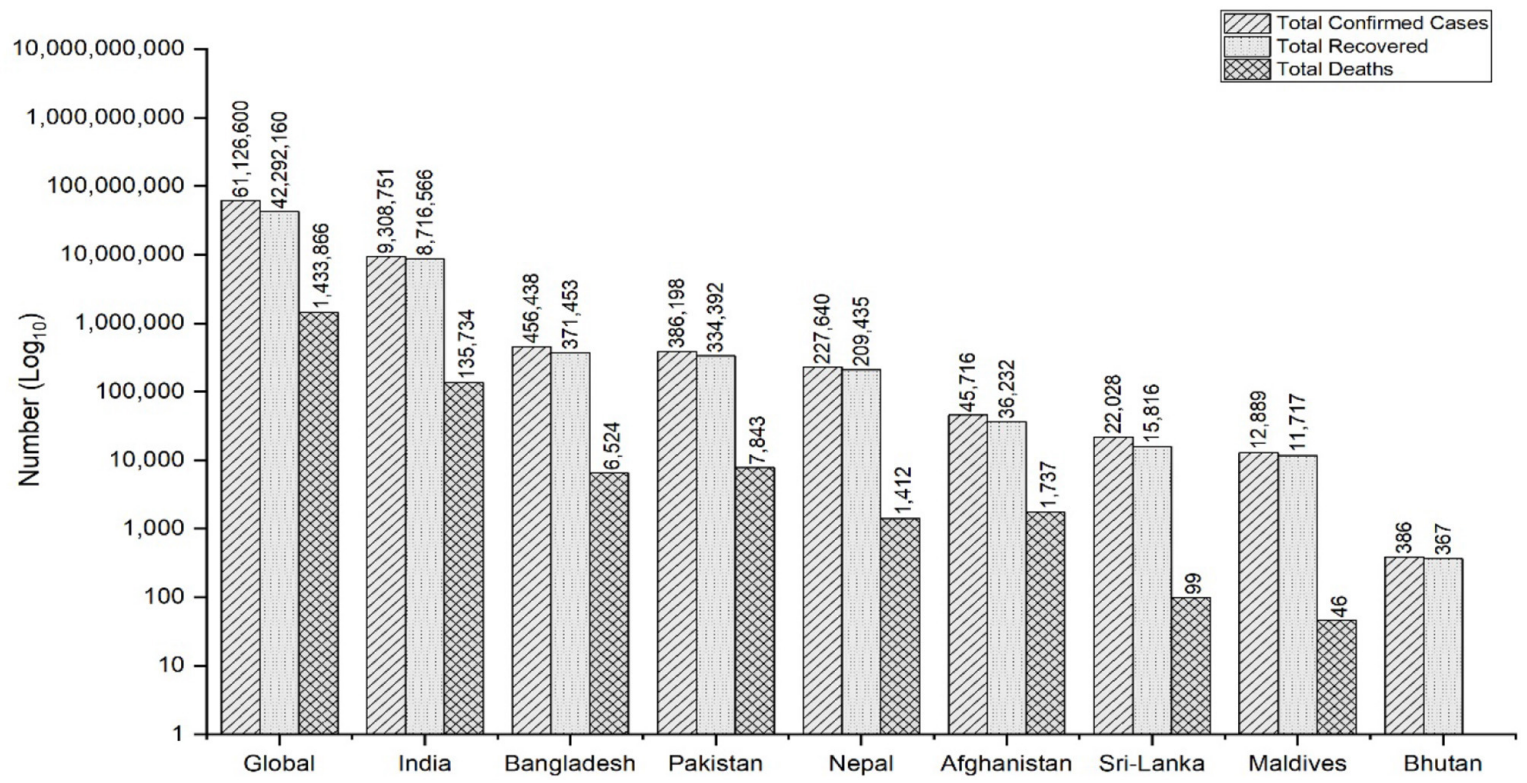
COVID-19 cases in SAARC member states.

Severe acute respiratory syndrome coronavirus 2 (SARS-CoV-2), which causes the coronavirus disease (COVID-19), was first identified in Wuhan, China, in December 2019 and later spread globally (1). The World Health Organization (WHO) declared the outbreak a Public Health Emergency of International Concern (PHEIC) on 30 January (2) and a pandemic on 11 March 2020 (3). COVID-19 has now affected over 218 countries and territories around the world, as well as two international conveyances (4). As of 26 November 2020, there have been 61,126,600 COVID-19 cases confirmed globally, with 1,433,866 (2.35%) deaths and 42,292,160 (69.19%) recovered cases (4). The highest number of deaths have been reported in the USA (13,197,307), followed by India (9,308,751) and Brazil (6,170,827) (4). The South Asian Association of Regional Cooperation (SAARC), a regional inter-government consortium of eight South Asian countries, which includes Nepal, India, Bangladesh, Pakistan, Maldives, Bhutan, Sri Lanka, and Afghanistan, comprises over 21% of the global population and is vulnerable to COVID due to multiple factors including poor health literacy, poor housing and living conditions, fragile health systems coupled with inadequately trained frontline health workers, poor quality of health care coupled with the poor diagnostic capacity of laboratory facilities and population of poor and migrant individuals (5, 6). As of 26 November 2020, Nepal holds the fourth position among the SAARC member countries in terms of total COVID infections and death cases, with India placing first and Bhutan placing last (Figure 1) (5).

## Situation of COVID-19 in Nepal

**Figure 2:**
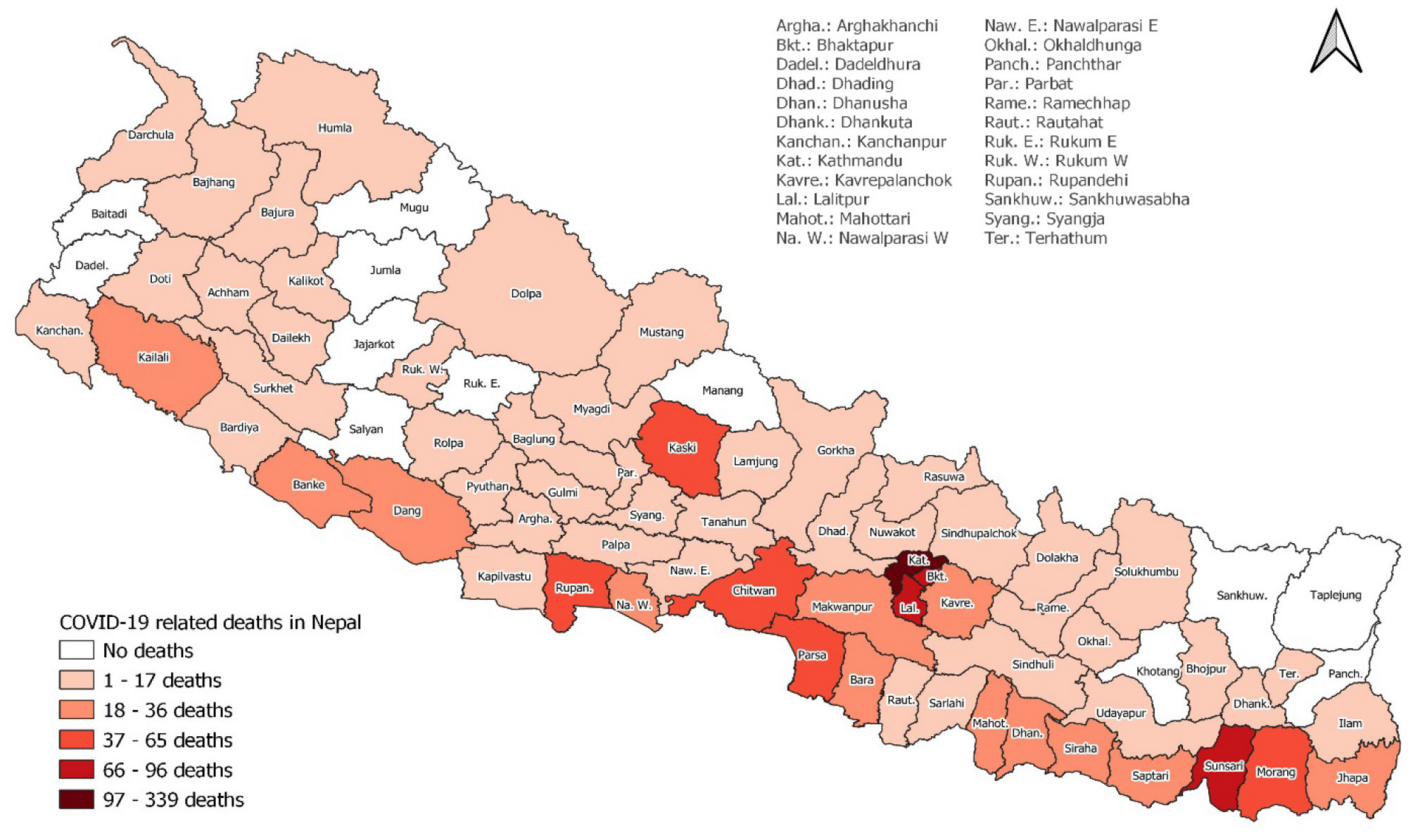
District-wise death cases of Covid-19 in Nepal.

Nepal adopted a federal democratic structure in 2017 and currently has seven provinces: Province No. 1, Province No. 2, Bagmati Province, Gandaki Province, Lumbini Province, Sudurpaschim Province and Karnali Province. Of the provinces, Bagmati Province currently has the highest number of COVID-19 cases and has reported the highest number of deaths (48.87%, *n* = 690). Out of 77 districts, the Kathmandu district reported the highest number of mortalities (24.00%, *n* = 339), followed by Lalitpur (6.8%, *n* = 96), Bhaktapur (6.30%, *n* = 89), and Sunsari (5.02%, *n* = 71) (Figure 2).

**Figure 3:**
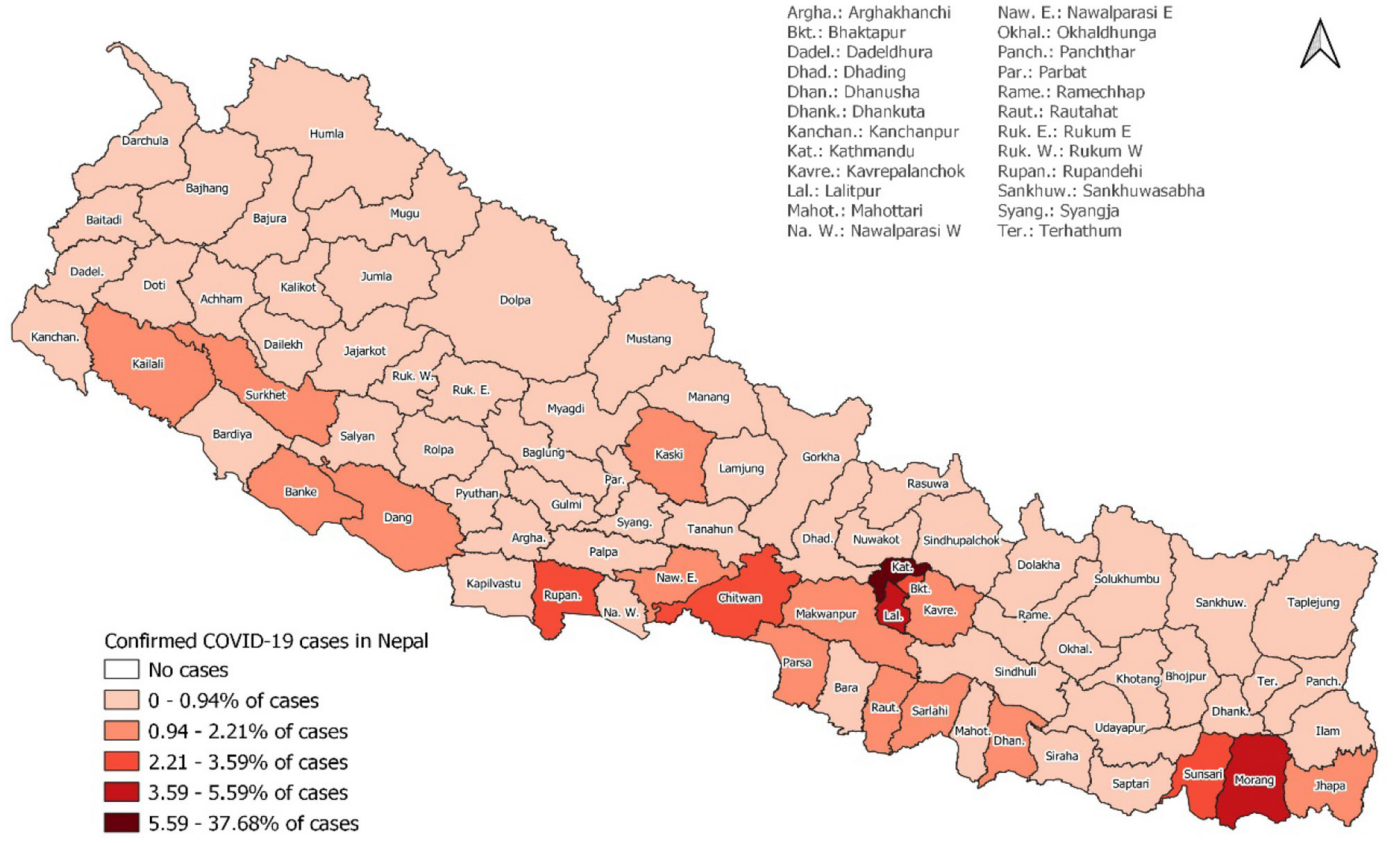
Cumulative cases of COVID-19 cases in different districts of Nepal.

The first positive case of SARS-CoV-2 in Nepal was reported in a Nepalese national who had returned to Kathmandu from Wuhan, China on 23 January 2020 (7). For several weeks after the first case which was reported in the last week of January 2020, Nepal did not witness an increasing number of COVID cases. However, since July and August 2020, Nepal experienced an unexpected surge of cases every day. As of 26 November 2020; 1,700,000 reverse transcription polymerase chain reaction (RT-PCR) tests have been performed in seven provinces, of which 227,640 have tested positive for SARS-CoV-2 (Figure 3).

**Figure 4:**
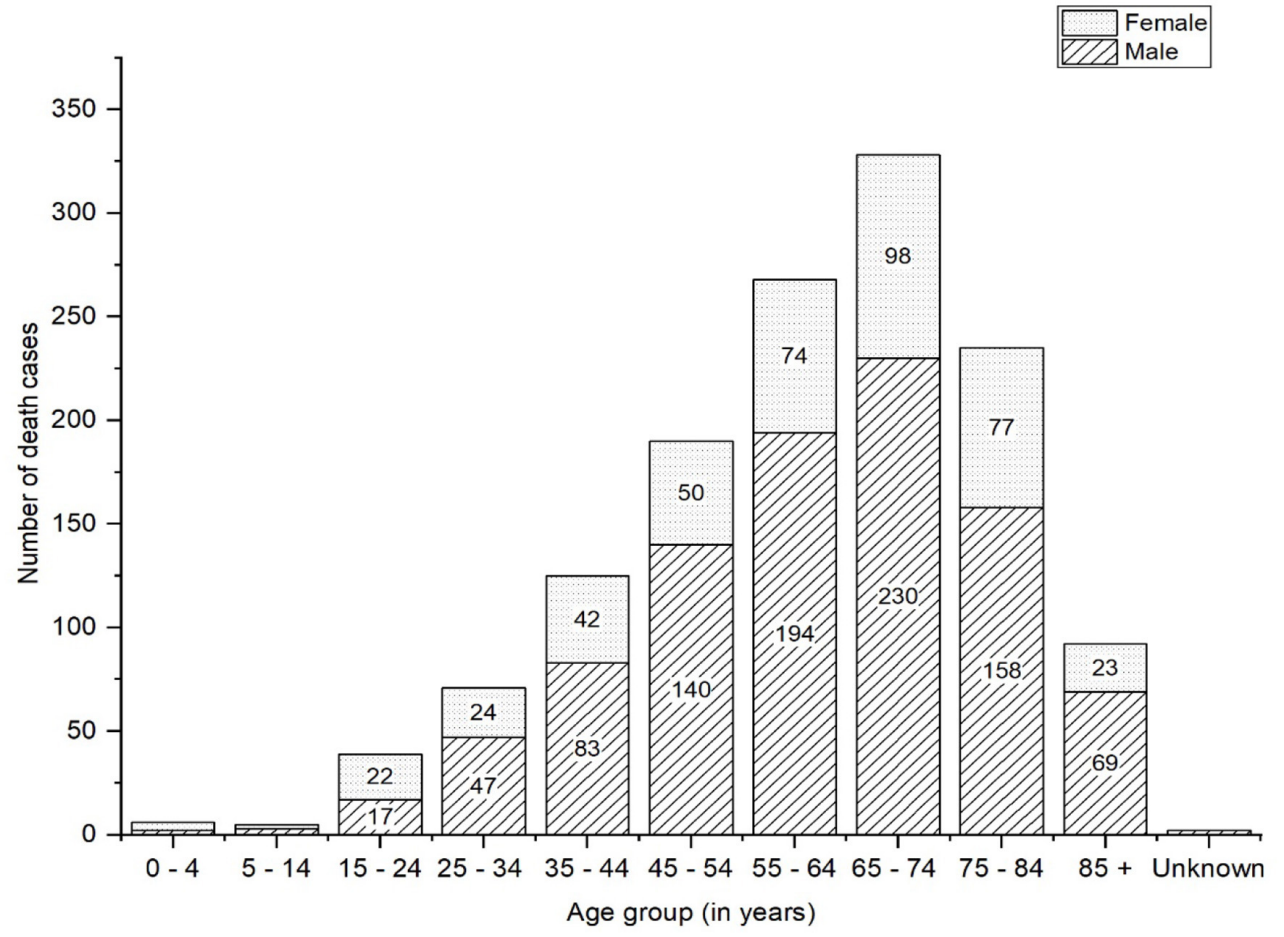
Age wise distribution of deceased COVID-19 patients.

Of the total positive cases, 209,435 have recovered, and 1,412 deaths have been confirmed. A total of 16,793 cases are active and under isolated surveillance, with more than 600 suspected cases currently in quarantine (8). As of 24 November 2020, deceased patients who were older than 54 years accounted for 67.82% (*n* = 923), while those aged 25–54 years accounted for 28.36% (*n* = 386), and of total deceased patients 69.43% (*n* = 945) were male (Figure 4). Patients older than 85 years have the highest case fatality rate (CFR) of 8.5% as of 24 November 2020 (9).

**Figure 5:**
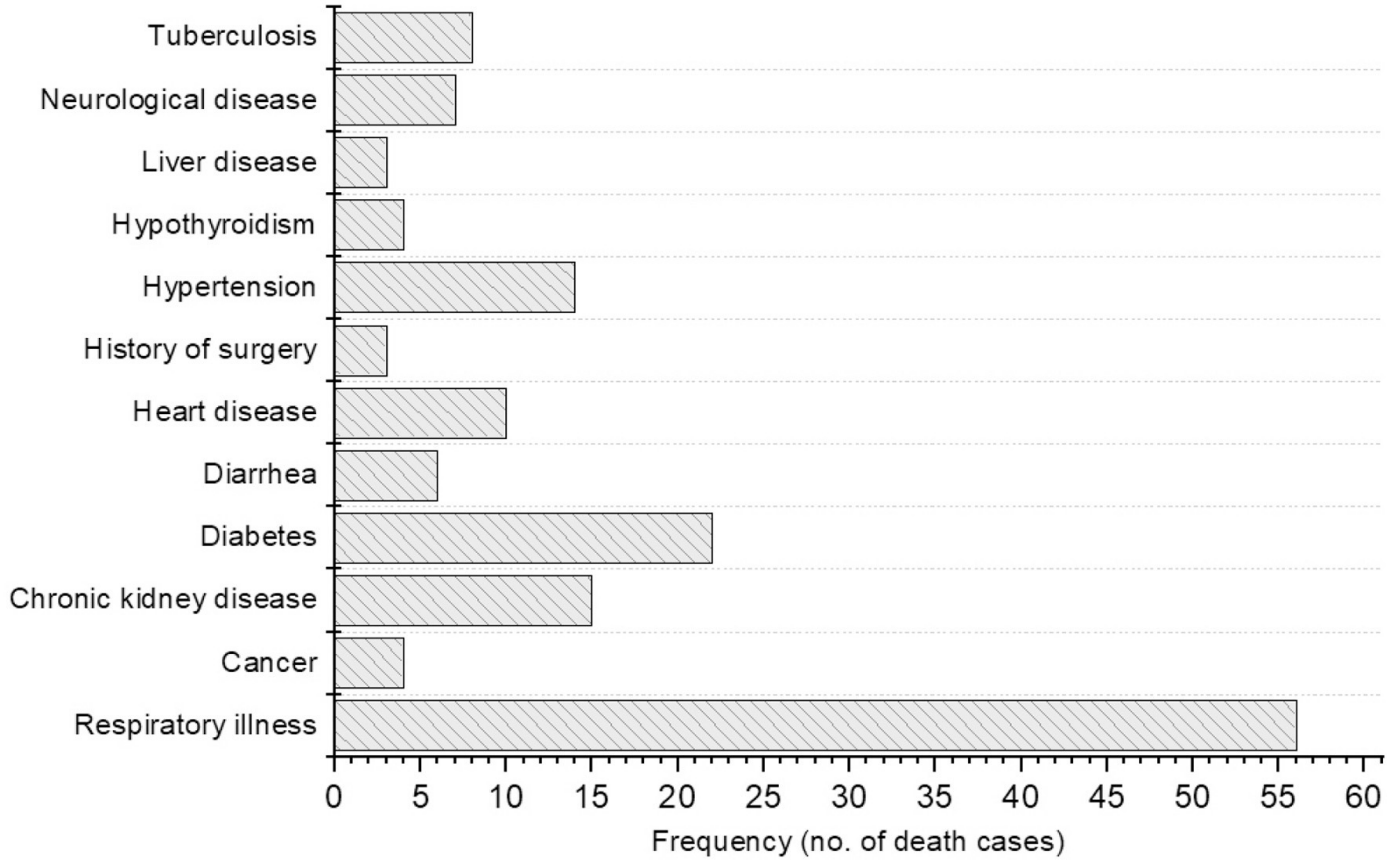
Other underlying illness in deceased COVID-19 patients.

The first death reported in Nepal was a 29-year-old pregnant woman. One deceased patient was an organ recipient, and three had a history of surgery. As of 16 August 2020, the Government of Nepal (GoN) has issued data of the underlying infections in deceased COVID-19 patients but later the GoN stopped to release the data on the underlying illness due to spiked cases of COVID-19 deaths. We have reported the available data of till mid-August to highlight the underlying illness among deceased COVID-19 patients in Nepal. The most common underlying conditions among the 104 deceased patients were respiratory illness (including asthma, pneumonia and other non-specified, 53.85%, *n* = 56), diabetes (21.2%, *n* = 22), chronic kidney disease (14.4%, *n* = 15), and hypertension (13.5%, *n* = 14) (Figure 5). Thirty-eight of the deceased patients had suffered from two or more comorbidities. Six patients died either in self-isolation or before they were able to reach a hospital and, therefore, other underlying illness could not be assessed.

As of 24 November, among all deceased patients, 895 (65.76%) patients had at least one pre-existing condition of comorbidity and such comorbidity was recorded apparently higher in older patients, highest comorbidity (26.82%, *n* = 240) being recorded in the age group 65–74 years (9).

**Figure 6:**
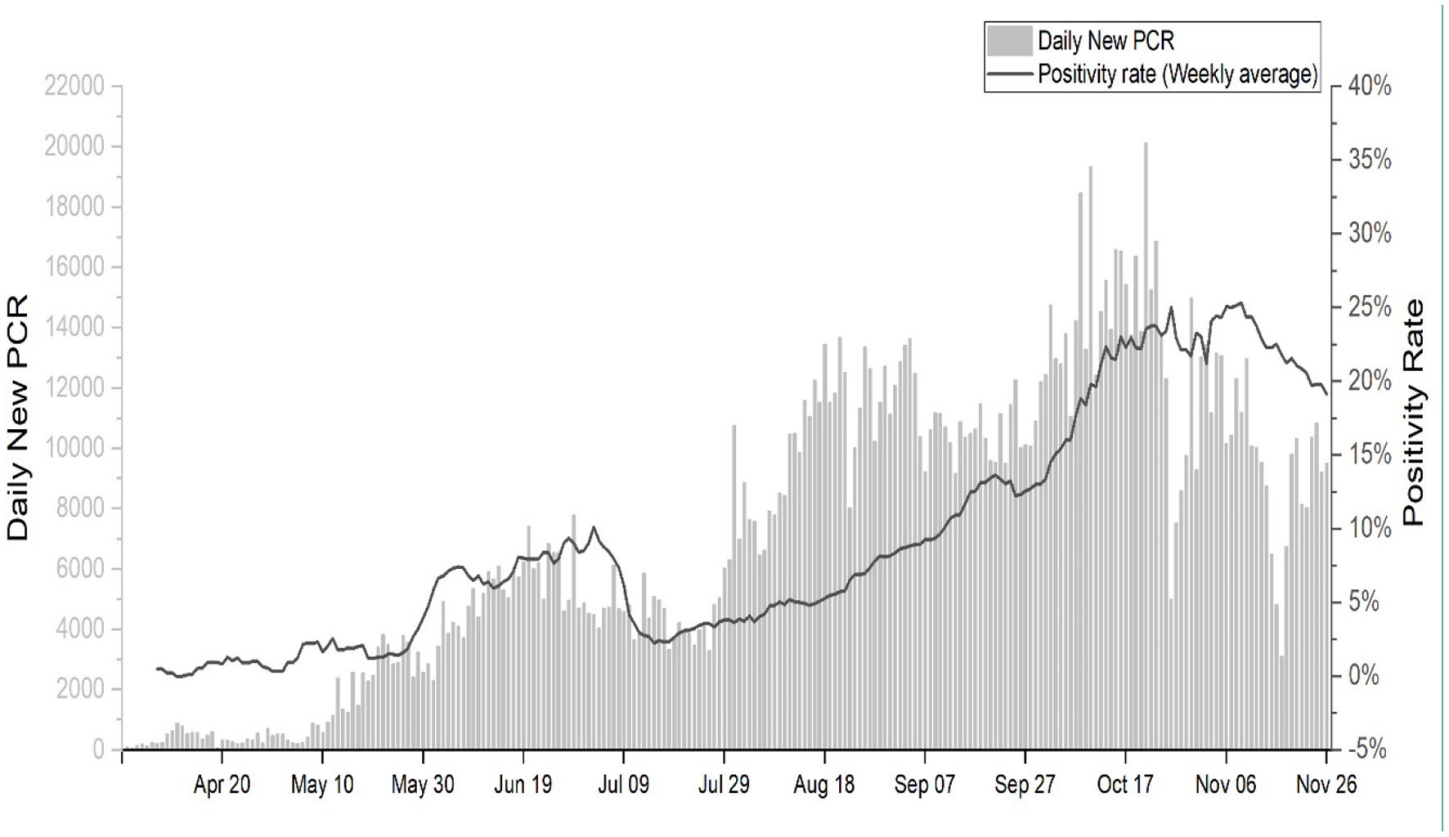
Trend of daily RT-PCR and positivity rate.

As the number of RT-PCR tests has increased (Figure 6), Nepal has witnessed increasing numbers of COVID cases, but the number of cases requiring specialized care such as intensive care units or life support with ventilators is also increasing every day (10). Several factors may have influenced the increased number of COVID cases in the country. First, the outbreak may be spiraling upward due to the cohabitation practices of the joint family system in Nepal, which is similar to Northern Italy (11). The southern plain region of Nepal-Terai, which has an open border with the northern part of India, a country that occupies the third position in terms of global COVID cases, has also been a hotspot; this area is the source of an influx of Nepalese migrants, which may have contributed to a higher number of COVID cases in this region. Despite continuous efforts, the Nepalese authorities have so far failed to systematically and effectively quarantine citizens returning from other countries, especially from India. Despite these facts, it is significant that most deceased patients had no travel history, and this suggests local community transmission of the disease.

## Pre-Existing Conditions and COVID-19

The preliminary analyses of data (16 August 2020) on underlying illness in deceased COVID-19 patients of Nepal showed nearly 87% of patients had pre-existing conditions i.e., non-communicable diseases (NCDs). The evidence shows that people living with NCDs are more vulnerable to severe illness or death as a result of infection with COVID-19 (12, 13). In Italy, among those COVID patients who died in hospitals, 68% had hypertension, and 31% had type-2 diabetes. In India, 30% fewer acute cardiac emergencies reached health facilities in rural areas in March 2020 compared to the previous year. In the Netherlands, the number of people newly diagnosed with cancer dropped by 25% as a result of lockdown measures imposed by the government. In Spain, among patients with severe symptoms, 43% had existing cardiovascular diseases (12). Bhatraju et al. (14) reported that majority of the COVID cases in the US had comorbid conditions, particularly NCDs such as diabetes (58%), kidney dysfunction (21%), asthma (14%), and chronic obstructive pulmonary disease (COPD) (4%). The same study reported that 33% of patients had more than one coexisting condition (14). Guan et al. recorded hypertension in 16.9%, diabetes in 8.2%, hepatitis B infection in 1.8%, chronic kidney disease in 1.3%, malignancy in 1.1%, and immunodeficiency in 0.2% of COVID patients in China (15). They also reported two or more comorbid conditions in 8.2% of patients with SARS-CoV-2 infection. These multi-morbid conditions were observed more commonly in COVID cases with severe conditions than in cases with mild or no symptoms (14, 16). We found a greater number of comorbidities correlated with greater disease severity in Nepalese COVID patients. The overall case fatality ratio (CFR) across all ages in Nepal has remained <1% (8, 17).

The world has witnessed discrepancies in the burden of COVID cases and the challenges of managing cases effectively and efficiently. Such discrepancies cannot be explained solely by current patterns of population age groups in countries like Nepal, where health care facilities have been heavily disrupted by the ongoing pandemic (11, 18, 19). The severity and mortality rate of COVID-19 has been increased by the presence of underlying comorbid conditions, mainly the NCD conditions such as hypertension, diabetes, cardiovascular diseases, respiratory diseases, cancer, kidney dysfunction, liver diseases, neurological disorder, autoimmune disorders, and other immunosuppressive conditions (20–22). Global evidence shows that severity and mortality have been reported at higher rates for male patients and older adults (18, 20, 22). Currently, available evidence shows that death occurs at an average of 17.8 days from the onset of COVID symptoms (23). Stories posted on social media platforms and media reporting of the situation have shown the miserable condition of health service delivery in Nepal, where people have been unable to access basic medicines or care in hospitals (particularly in areas with protracted lockdowns). Furthermore, it has been reported that patients who failed to present COVID reports were not allowed to receive inpatient care and that some patients lost their lives in search of a health care center or as a result of not having access to the ambulance services necessary to reach health facilities. WHO states that most people requiring treatment for NCDs like cancer, cardiovascular disease, and diabetes have not been able to receive health services and essential NCD medication as a consequence of the pandemic and the resultant lockdown imposed by central and local governments (12). This situation warrants urgent action to introduce innovative and alternative approaches to providing NCDs services. WHO also recommends including NCDs in the national response to and preparedness plans for managing the pandemic (13).

## The GOvernment of Nepal's Response to the COVID-19 Pandemic

Similar to many other countries, Nepal has massively endured unprecedented COVID pandemic with huge economic loss (24). To stop the rapid spread of SARS-CoV-2, the GoN has taken actions such as stay-at-home and mass quarantines (25). Movement restriction of people appears the most efficient non-clinical intervention to contain the spread of COVID, especially in resource-limited nations such as Nepal. At present, COVID cases are across the country because of inadequate implementation of the strategies and policies required for management of the overwhelming pandemic. However, the GoN is putting constant effort to curb the rapid transmission of SARS-CoV-2 at the community level.

### Testing Strategies

In the earliest weeks of the COVID-19 pandemic, the health surveillance desk at Tribhuvan International Airport—the only international airport in the country—was initially authorized to screen all incoming passengers from affected regions around the globe (26). The government, under the leadership of the home minister, formed a patient receiving team to transport the suspected patients to hospitals designated to receive COVID patients (27). RT-PCR tests were performed for quarantined individuals who displayed suspected COVID symptoms. Uninterrupted treatment services were prioritized by setting up twenty-five-bed hospitals. Additionally, the government of Nepal has set up 36 COVID dedicated hospitals categorized into three levels: 16 Level 1 hospitals responsible for treating mild cases, 16 Level 2 hospitals for treating moderate cases, and four Level 3 to provide advance and specialized care for severe cases (28). All confirmed cases were shifted immediately and managed at COVID hospitals until they tested negative according to RT-PCR tests. In the meantime, the government ensured uninterrupted treatment services to non-COVID patients with life-and-limb-threatening conditions or injuries such as abscesses, acute pain, heart failure, acute exacerbation of COPDs, haemodialysis and ketoacidosis, among others (28). The GoN designated the Sukraraj Infectious and Tropical Disease Hospital (SITDH) in Kathmandu as the primary hospital for COVID treatment, along with the Patan Hospital and the Armed Police Forces Hospital. The government also designated specific spaces to be used for quarantine purposes throughout the country. The ground crossing points of entry at the Nepal-India border and the Nepal-China border were tightened in March 2020.

### Contact Tracing

Effective systems to trace the contacts of those people who have come into contact with carriers of COVID-19 is essential to stop the rapid spread of the disease (29). With this in mind, the GoN has set up a system to screen individuals arriving in Kathmandu from other parts of the country or overseas by road or by air. Symptomatic passengers were taken directly to designated COVID hospitals and admitted, tested and treated appropriately. In contrast, asymptomatic passengers were kept in dedicated quarantine or advised to follow strict home quarantine and self-isolation and to avoid non-essential travel and community contact. Despite these regulations, there was no mechanism in place to monitor home quarantine and self-isolation.

In May 2020, the government formulated the Health Sector Emergency Response Plan (HSERP) to manage the spread of COVID-19. The plan suggested forming Case Investigation and Contact Tracing Teams (CICTTs) at the local level, which would include members from the public health, laboratory, nursing, local council, administration and security sectors (30). To date, most of the councils in the country have formed CICTTs. The main functions of the CICTTs are to ensure trained human resources at all levels, follow standard operating procedure (SOP) for case investigation and contact tracing, mobilize Female community health volunteers (FCHVs) at community levels, perform rapid epidemiological investigations of clusters at risk for COVID-19, and ensure necessary resources and protective measures to all care providers according to the estimated level of risk (31). These are essential functions for COVID-19 prevention and control and are outlined clearly as core functions of CICTTs. However, the key question remains whether these CICTTs are functioning effectively to trace contacts and ensure COVID testing for people at risk. So far, no detailed information or updates are available regarding the effectiveness of these CICTTs; however, several media reports suggest that CICTTs across the country have been inefficient and that this is a key area requiring the careful attention of the MoHP, to fight the spread of COVID-19 in Nepal.

### Management of Quarantine and Isolation Centers

Media reports also show that quarantine facilities are becoming breeding centers for COVID-19 due to dangerous crowding, lack of facilities for sanitation and hygiene, poor residential environments (such as detainees sleeping on benches in communal rooms), poor medical care and social support and lack of nutritious food services (32). Institutional quarantine facilities have been arranged at schools, campuses, hostels, hotels and other accommodating facilities with the coordination of local government, and isolation facilities have been organized by the provincial governments and various public and private hospitals across the country. The mechanism developed by the government for monitoring the quarantine is ineffective in many places due to lack of proper coordination between stakeholders.

### Public Information and Awareness Campaign

Disseminating correct information and education to the public is an effective measure to control and prevent the spread of any disease, especially in the time of the pandemic (28). The prevention of highly contagious and infectious disease is critically important (24). The Nepalese government, in collaboration with a range of sectors (both private and public) and other stakeholders, began developing and implementing information and awareness programs in the fight against COVID-19. Several public awareness campaigns designed to break the transmission chain were issued through text, audio and video platforms such as newspapers, flyers, radio and television. Despite these efforts, the potential risk of coronavirus transmission at the community level is not being taken seriously in Nepal, and this may be due to low health literacy level. Such negligence has been observed in media reports and social media stories that describe people found not following physical distancing practices in open places; for example, community dwellers observed selling and purchasing groceries in open markets without practicing physical distancing. The condition could worsen if the government fails to implement basic safety protocols; this could be achieved through awareness campaigns designed to encourage wearing masks properly in public places, using hand sanitiser, washing hands frequently, avoiding crowds and maintaining at least three meters of physical distance. These measures are needed especially considering upcoming festivals, which are celebrated by a large population of Hindu communities. Social media reports also show that some non-allopathic practitioners are openly distributing ineffective remedies and creating a false sense of security among the community. Local government bodies need to monitor these claims closely and take action against the spread of misinformation about treatments for COVID-19 (33).

Similarly, various protests for and against the ruling government have posed a significant challenge to authorities' efforts to contain the outbreak. Hundreds of young people chanting “Enough is Enough” have been descending on the streets of major cities across the country demanding a better and effective response from the government to curb the COVID-19 outbreak (34). While such protests and demonstrations would be important to compel the government for its transparency, accountability, and response to people in terms of prevention and control of COVID-19, demonstrators and protestors need to comply with public health preventive advice such as using face masks properly and maintaining physical distancing, as suggested by WHO guidelines.

### Nation-Wide Lockdown

On 2 March, in response to the ongoing pandemic, Nepal's government suspended the visa-on-arrival scheme for citizens of China, Hong-Kong, Japan, Italy and Iran, which are severely affected by COVID-19. The scheme was updated on 11 March and suspended for all nationals including non-residential Nepalese (NRN). On 18 March, the GoN planned to ban all passengers, including Nepalese nationals, from entering Nepal from European Union member countries, the United Kingdom, West Asia, the Gulf countries, South Korea and Japan; this ban was effective from midnight of 20 March 2020. On the same day a high-level coordination committee to fight against COVID-19, led by the defense minister, decided on the postponement of Secondary Education Examination (SEE) and university exams; the National Examinations Board (NEB) imposed a ban on all gatherings of more than 25 people. On 20 March, the government announced more measures to restrict outbreaks, including temporary bans on all flights, long-distance transportation across the country and all non-essential services. The government has included the following services in the essential services category: telephone and communication, transportation, civil aviation and airport, government press, defense affairs relating to arms, the production of military goods, internal security, drinking water, electricity, the residence of tourists, petroleum products, health and medicine and banking and insurance.

Amid concerns about SARS-CoV-2 spreading in the community, the government imposed a nation-wide lockdown effective 24 March, hours after the second COVID-19 case was confirmed. This measure was intended to last a week but was later extended to about four months. All national and international flights ceased, and all public and private vehicles were banned from March 22, 2020 except for those with prior permission from local authorities, those belonging to security forces, health workers and ambulances. Groceries and pharmacies operated during times specified by local authorities, while all other workplaces, government offices, school and universities were closed. Anyone who defies the government order is arrested under the Infectious Disease Control Act. According to the Act, violators are liable for a jail term of a month or hundred Nepalese rupees as fines, or with both penalties.

Recently, the Nepalese government decided to end the four-month-long lockdown effective from 22 July, with few restrictions, and to allow all domestic and international airlines to resume from 17 August 2020; however, it has again extended the restriction till 31 August 2020. In the context of celebrating “Visit Nepal 2020,” the GoN also allowed hotels and restaurants to resume from 30 July while continuing all provisions related to COVID prevention, control and treatment strategies. Although the federal government has lifted the lockdown, some provincial and local authorities have re-imposed lockdown and other strategies to stop the further spread of SARS-CoV-2 in community. Some of these strategies include odd-even number vehicle movement, a complete prohibition of people from other districts, sealing commercial areas in some cities and ramping up contact tracing and testing at all levels of the government. Due to the second surge of COVID-19 in Nepal, province and districts are set to complete lockdown per the risk level.

### Economic Support Package

Lockdown has impacted every sector of the country and disproportionately affected the poor, daily wage earners, and other marginalized groups in rural areas who have access to food and other services only through day-to-day basis work. In response, the government has established the “COVID-19 Prevention, Control and Treatment Fund” to which hundreds of institutions, business firm and individuals have made contributions. On 17 July, Nepal Rastra Bank, the central bank of Nepal, unveiled relief packages (35) through its annual monetary policy to mitigate the economic effects of COVID-19. These packages comprise an extension of loan repayment deadlines, refinance facilities, grace period extension for infrastructure projects and targeted lending in productive sectors at cheaper rates. On 27 July the bank categorized various sectors into three groups based on the level of impact caused by COVID-19: highly-affected, semi-affected, and least-affected (36).

Similarly, the government and other stakeholders have also introduced a relief package for laborers, poor and marginalized people, as well as distributing urgent foodstuffs; however, the implementation of such relief packages is not happening effectively due to unavailability of data on poor households, a lack of proper coordination among the involved stakeholders and the lack of a monitoring mechanism for distribution packages. Furthermore, the country's resources for curtailing the pandemic are being challenged by flooding and landslides in Hilly and Terai regions of the country as the monsoon season enters its peak. These natural disasters have affected hundreds of households and family, leaving many people homeless or living in emergency shelters, which are congested; thus, people can hardly maintain the recommended social distance, and many cannot afford the supplies necessary for good sanitation practices.

## Opportunities for the Government of Nepal

**Figure 7:**
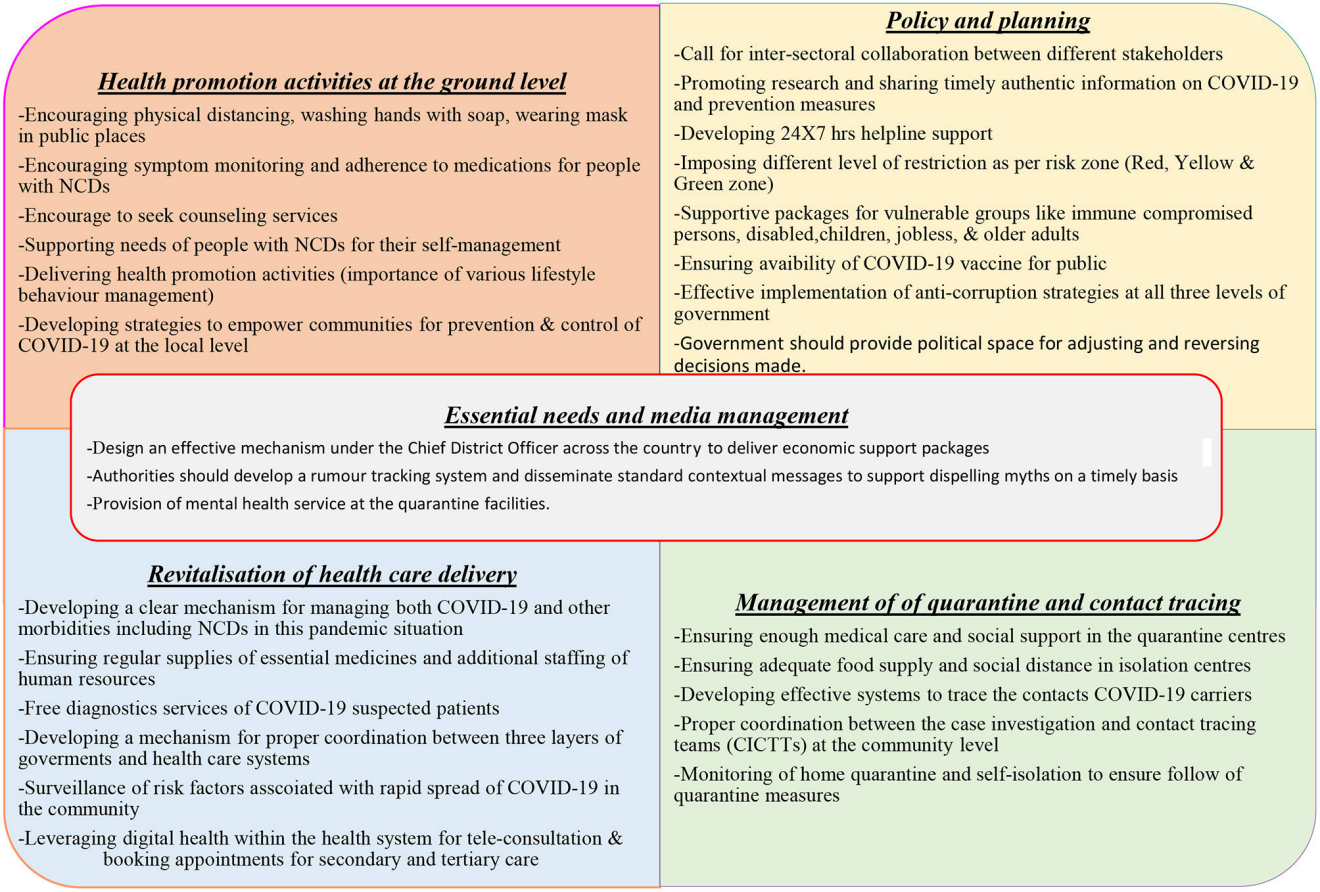
Diagrammatic presentation showing the priorities areas for the improvement of COVID19 management.

We describe the priorities areas for the GoN (Figure 7) that are essential to overcoming and managing COVID.

### Leadership and Management

In any uncertain situation, the population relies completely on leaders who are in a position to make crucial decisions during a challenging time. The government has created different committees and task teams for preparedness and response to COVID-19, but these committees have been criticized for not being able to perform preventive measures. In the current situation, the Nepalese leadership has been entangled in digressive topics: whether or not to accept the Millennium Challenge Corporation grant provided by the US government, whether Lord Ram (a Hindu God) was born in India or Nepal, whether new vehicles should be purchased for ministers and whether the ruling party prime minister should change. Moreover, the COVID-19 crisis has exacerbated several weaknesses including a scandal related to the procurement of Chinese personal protective equipment (PPE) and newly purchased but malfunctioning polymerase chain reaction (PCR) machines, delays in receiving expert opinions and expert disagreements with government decisions, understaffed and under-resourced public health care systems, a lack of media management, insufficient supplies and resources, poor planning and inter-sectoral collaboration and support, mismanaged quarantine, isolation centers, and testing processes and a lack of coordination between the three layers of the government system—federal, provincial, and local governments—in the management of this overwhelming condition.

The government should avoid the temptation of heroic leadership and instead need to listen to experts, implement anti-corruption strategies more effectively at all three levels of government (federal, provincial, and local level) and co-produce and implement policies for the effective management of an unpredictable condition that requires ultimate sacrifice from all politicians. It is also crucial that the government provide political space for adjusting and reversing decisions made. Government authorities need to engage the private sector in COVID management (37), which is not happening, with a few exceptions.

### Essential Supplies, Media, and Quarantine Management

The lockdown imposed by authorities caused panic among the most marginalized and disadvantaged populations, particularly daily wage workers, older adults, people affected by natural disasters, people with disabilities and widow(er)s. To avoid such situations, the government should design an effective mechanism under the Chief District Officer across the country to deliver economic packages to the most affected population at the smallest administrative level which is ward area of each local government. The National Health Education Information Communication Center has developed standardized messages at the federal, provincial and local levels of government; however, misinformation and fake news about COVID-19 is spreading through social media platforms and online news portals. Authorities should develop a rumor tracking system and disseminate standard contextual messages to support dispelling myths on a timely basis. Despite being a resource-constrained country, Nepal is trying its best to improve the conditions of its quarantine facilities. The poor quality of quarantine centers has led to rumors and discrimination against people who have had stayed there; the government must ensure that these facilities provide all basic amenities, including nutritious food, gender-friendly sleeping areas and clean toilets (38). Similarly, the provision of mental health service at the quarantine facilities would be a great support for people who are staying away from their family members (39). Moreover, separate isolation facilities for asymptomatic and symptomatic people, as well as a facility to refer patients to designated COVID-19 hospitals, would increase the smooth function and use of the quarantine facilities.

### The Revitalisation of Health Care Delivery

The pandemic has exposed the long-standing fragile health care system of Nepal. The country has been battling the worst health effects while responding to COVID-19 with an under-resourced and understaffed health care system (40), and this has created a public demand throughout the country for quality and timely health services. Currently, secondary and tertiary health systems in Nepal are overwhelmed with the management of COVID-19 cases and, at the same time, the priority to address the needs of other infections and NCD has not been prioritized due to a lack of quality human resources, health system capacity and significant resource constraints. The Nepalese authorities need to develop a clear mechanism and system for managing both COVID-19 and other morbidities; compiling a list of operational COVID-19 and non-COVID-19 hospitals will enable people to receive health care services. Similarly, the government must strengthen primary health care services through regular supplies of essential medicines and extra staffing of human resources for health services; these efforts may reduce the patient burden on secondary and tertiary hospitals (41).

Moreover, the local government should manage ambulances with trained medical personnel to take the patients to the respective hospital so that patients can receive timely treatment. Similarly, leveraging digital health within the health system to provide teleconsultation and online appointment booking for secondary and tertiary care would be a benefit for patients. Local governments should not depend on every decision from a federal government, and it is high time that local governments ramp up contact tracing and testing by recruiting volunteers and installing PCR machines at the local levels. Finally, all three levels of government should develop and provide mental health advice and support platforms to help people to cope with the economic downturn, uncertain situations and the isolation, loneliness and anxiety that are creating a syndemic pandemic (41). It is equally important to consider the vulnerable and hard-to-reach rural communities as a part of the national response against the COVID.

## COVID-19 Vaccination Strategy and Adaptation in Nepal

The scientists and researchers have worked around the clock with an unpredicted speed and finally it bought a ray of hope to fight the pandemic plight faced by global population. As said by WHO “A vaccine alone will not end this pandemic” means initial supply of vaccines will be prioritized for the high-risk and vulnerable population because of limited capacity of production and supplies in this unpredicted time. In the meantime, GoN should need to make a strategic plan and direct all energy and resources to prepare the country ready for availability of vaccines. Government should start working with GAVI COVAX Advance Market Commitment (AMC) to (42) ensure that vaccines are secured for Nepal with an effective delivery mechanism. Moreover, the country should start preparatory work (43) with experts of international and national levels, academics/researchers, international and national organizations, provincial and local governments to develop the protocols, mechanism for effective distribution and infrastructures required for rolling out the vaccines in timely and equitable way as soon as vaccine becomes available.

## Conclusion

Along with the surge of COVID cases in Nepal, fatal cases are increasing, especially after the 4-month nation-wide lockdown was lifted. Numerous cases are being reported in southern Nepal, which shares a common border with India, as well as in other major cities in the country. Although older adults are considered more vulnerable to the infection of SARS-CoV-2, recent data from Nepal shows high case fatalities among younger patients as well. Respiratory illness, such as asthma and pneumonia are the most prevalent comorbidity associated with COVID mortality in Nepal. Executing the SOP for quarantine facilities effectively, increasing testing and effective contact tracing, and providing uninterrupted treatment for conditions other than COVID-19 may yield efficacious control of the pandemic in a resource-limited country like Nepal; more appropriate and effective measures for containing the rapid spread of SARS-CoV-2 are urgently necessary. A health literacy campaign and a clear strategy to care for older people and those with existing conditions like NCDs may also contribute to the effective management of the ongoing pandemic. As suggested by Torres et al. (44), this pandemic has worsened social equality in low-income countries including Nepal, and this needs to be addressed mainly by prioritizing marginalized and vulnerable populations. As reported in Nepal (37) and elsewhere (38) some patients show viral shedding 14 days after an initial positive test and may pose a risk of transferring the infection to a cluster or the broader community, therefore extending the current quarantine period of 14 days is recommended to minimize the spread of the virus. The Nepalese government should pay special attention to mass testing and quarantine management via the effective coordination of its three tiers of government (federal, provincial and local) to control rapidly spreading SARS-CoV-2 in the local communities.

## Author Contributions

BR and UY designed the study. GS collected the data. BR, SK, and AP analyzed the data. AP, UY, and BR drafted and edited the manuscript. LR, SM, RP, BL, and SKM contributed significantly. All authors read and approved the final version of manuscript.

## Conflict of Interest

The authors declare that the research was conducted in the absence of any commercial or financial relationships that could be construed as a potential conflict of interest.
